# The Promise of Retinoids in the Treatment of Cancer: Neither Burnt Out Nor Fading Away

**DOI:** 10.3390/cancers15143535

**Published:** 2023-07-08

**Authors:** Yuya Nagai, Alexander J. Ambinder

**Affiliations:** 1 Department of Hematology, Kobe City Medical Center General Hospital, Kobe 650-0047, Hyogo, Japan; 2 Department of Oncology, Sidney Kimmel Comprehensive Cancer Center, Johns Hopkins University School of Medicine, Baltimore, MD 21231, USA

**Keywords:** retinoid, ATRA, tamibarotene, RARα, CYP26, differentiation therapy, APL, non-APL AML

## Abstract

**Simple Summary:**

In the realm of cancer treatment and prevention, the application of retinoids has led to unparalleled successes and humbling disappointments; all-trans retinoic acid (ATRA) has transformed APL (acute promyelocytic leukemia) into an eminently curable disease but has demonstrated little efficacy in acute myeloid leukemia (AML) more broadly. As a cancer prevention strategy, vitamin A supplementation was ineffective and appeared to increase lung cancer incidence, underscoring the complex relationship between retinoic acid signaling and carcinogenesis. This review aims to provide a survey of our current understanding of retinoic acid homeostasis and signaling, to reflect on prior successes and failures to deploy retinoids in the treatment and prevention of cancer, and to explore the unfulfilled potential of retinoids in cancer. New biological insights relating to the regulation of retinoid homeostasis and the discovery of biomarkers of retinoid sensitivity in subsets of non-APL AML have revived enthusiasm for the therapeutic potential of synthetic retinoids, and thus warrant a reacquaintance with retinoid biology.

**Abstract:**

Since the introduction of all-trans retinoic acid (ATRA), acute promyelocytic leukemia (APL) has become a highly curable malignancy, especially in combination with arsenic trioxide (ATO). ATRA’s success has deepened our understanding of the role of the RARα pathway in normal hematopoiesis and leukemogenesis, and it has influenced a generation of cancer drug development. Retinoids have also demonstrated some efficacy in a handful of other disease entities, including as a maintenance therapy for neuroblastoma and in the treatment of cutaneous T-cell lymphomas; nevertheless, the promise of retinoids as a differentiating therapy in acute myeloid leukemia (AML) more broadly, and as a cancer preventative, have largely gone unfulfilled. Recent research into the mechanisms of ATRA resistance and the biomarkers of RARα pathway dysregulation in AML have reinvigorated efforts to successfully deploy retinoid therapy in a broader subset of myeloid malignancies. Recent studies have demonstrated that the bone marrow environment is highly protected from exogenous ATRA via local homeostasis controlled by stromal cells expressing CYP26, a key enzyme responsible for ATRA inactivation. Synthetic CYP26-resistant retinoids such as tamibarotene bypass this stromal protection and have shown superior anti-leukemic effects. Furthermore, recent super-enhancer (SE) analysis has identified a novel AML subgroup characterized by high expression of RARα through strong SE levels in the gene locus and increased sensitivity to tamibarotene. Combined with a hypomethylating agent, synthetic retinoids have shown synergistic anti-leukemic effects in non-APL AML preclinical models and are now being studied in phase II and III clinical trials.

## 1. Introduction

The remarkable success of all-trans retinoic acid (ATRA) therapy in APL has come to epitomize the importance of retinoic acid signaling in carcinogenesis and the anti-cancer potential of retinoids. Nonetheless, efforts to replicate the success of ATRA in APL with other non-APL leukemias and malignancies have led to disappointing results [1,2,3]. Recent improvements in the understanding of retinoic acid biology, the development of synthetic retinoids, and an improved capability to identify subsets of non-APL AML with increased sensitivity to retinoids have revived hopes for the successful use of retinoids beyond APL [4,5,6,7,8]. In this article, we review the history of the introduction of ATRA into clinical use, summarize recent developments in the understanding of the role of retinoic acid signaling in non-APL AML, and discuss promising strategies to successfully target retinoic acid pathways in the treatment of non-APL AML.

## 2. The History of Retinol, Health, and Cancer

Vitamin A is a fat-soluble vitamin essential in orchestrating embryogenesis, cellular differentiation, and proliferation [9]. Much of what is known about the biological role of retinol has been learned from the effects of its absence. One of the most visible adverse effects of vitamin A deficiency (VAD) is xerophthalmia or night blindness. Records from ancient Greece, Egypt, and China have all documented animal liver as an effective remedy for night blindness, likely owing to its high concentration of vitamin A stores [10]. Nutritional studies in the early 1900s first identified a fat-soluble dietary factor, Factor A, present in butter and eggs and necessary for rats to continue growing. Today, VAD remains a significant cause of morbidity and mortality worldwide. In population studies, a deficiency of vitamin A has been associated with xerophthalmia, stunted growth, anemia, dermatologic manifestations including poor wound healing, impaired immune function, and increased maternal and childhood mortality. In populations with a high prevalence of VAD, vitamin A supplementation has been demonstrated to reduce early childhood mortality. VAD has therefore been targeted for elimination in an extensive public health campaign by the World Health Organization and the United Nations International Children’s Emergency Fund (UNICEF).

The potential role of vitamin A in oncogenesis was first suggested by animal studies in the mid-1920s, though the claim was met with skepticism, and data from different groups were conflicting [11]. Animal studies continued, with one notable study in 1959 showing a higher incidence of epithelial tumor production in mice treated with a carcinogenic-treated cheek pouch and a diet deficient in vitamin A, compared to control mice who received the carcinogenic treatment to the cheek pouch but otherwise had a vitamin-A-sufficient diet [12]. Then, in the 1970s, there were a series of both retrospective and prospective epidemiologic studies that found an inverse relationship between serum vitamin A concentration and cancer incidence, particularly with respect to lung cancer [13]. These studies sparked tremendous enthusiasm to understand the biology of retinoids and to harness them for the prevention and treatment of cancer. In the case of acute promyelocytic leukemia, these efforts have led to remarkable success and the development of retinoids as a model for targeted cancer therapy; however, the outcomes of studies using retinoids as cancer prophylaxis and in non-APL acute myeloid leukemias (AML) have been sobering, underscoring the complex role of retinoid metabolism in cancer biology.

## 3. Retinoids

Vitamin A metabolism is complicated by the diversity of forms that it takes in the body. Animals cannot synthesize vitamin A and thus must ingest retinol, the alcohol form of vitamin A, or provitamin A carotenoids such as beta-carotene. Enzymes in the liver can esterify them for storage, or they may be bound by retinoid-binding proteins for transport to target organs, where they are converted into retinal (retinaldehyde) by retinyl ester hydrolase and then into retinoic acid, the most active form of vitamin A, by retinaldehyde dehydrogenase [14]. ATRA is the most active form of endogenous vitamin A; however, numerous other isoforms, including 13-cis retinoic acid (Isotretinoin) and 9-cis retinoic acid, have varying degrees of affinity for the various retinoic acid receptors (RARs) [10]. In addition, some forms can bind RXR, in which case they are referred to as rexinoids. CYP26 then catabolizes retinoids into inactive metabolites, namely, 4-hydroxy retinoic acid and 4-oxo retinoic acid.

## 4. Retinoid Signaling

ATRA controls gene expression via the transcriptional activation of nuclear retinoic acid receptors (RARs) [15]. RARs form a ligand-regulated nuclear receptor superfamily responsible for regulating transcription. RARs consist of three isotypes, including RARa, RARb, and RARg, which are expressed by varying degrees across tissues and developmental stages. RARa is highly expressed in the developing and adult nervous system, lung, spleen, and hematopoietic cells in the bone marrow [1]. RARb is expressed in the lung, esophagus, and skin, whereas RARg is predominantly expressed in skin, skeletal muscle, and adipose tissue. RARs act as a nuclear transcription factor with the retinoid X receptor (RXR) cofactor. RARs form heterodimers with RXR and bind to specific motifs called retinoic acid responsive elements (RAREs) within the promoter of target genes. In the absence of a ligand, RAR/RXR interacts with the corepressor proteins, the silencing mediator of retinoid and thyroid hormone receptor (SMRT) and the nuclear receptor corepressor (NCoR). These corepressors, in turn, recruit histone deacetylase (HDAC), which triggers the chromatin to assume a transcriptionally inactive conformation. ATRA binds to the RAR and causes a conformational change of the ligand-binding-domain (LBD), leading to the receptor’s dissociation from corepressors and the promotion of the interaction with co-activators, including histone acetyltransferase (HAT). Thus, the binding of ATRA promotes the transcriptional activation of target genes and differentiation [16]. In the case of RARa, many of its target genes, including C/EBPepsilon, PU.1, and HOX proteins, are involved in myelopoiesis. RARa signaling is therefore involved in orchestrating hematopoietic stem cell quiescence, self-renewal, and differentiation into myeloid hematopoietic cells [1,17,18,19].

## 5. The Unparalleled Success of Retinoids in the Treatment of Acute Promyelocytic Leukemia

APL is a distinct subtype of AML characterized by the proliferation of abnormal promyelocytes and typically a severe coagulopathy accompanied by disseminated intravascular coagulation (DIC) or hyperfibrinolysis. Historically, chemotherapy alone exacerbates bleeding tendency, leading to a high incidence of early hemorrhagic death, and achieved cure in only 35% to 45% of patients with APL [20]. The discovery in the 1980s that retinoic acid induces the terminal differentiation of APL cells in vitro [21] and a clinical response with the differentiation of APL cells and improved coagulopathy in patients [22,23,24] promoted clinical trials of ATRA as a targeted therapy for the treatment of APL. The first documented use of retinoids in the treatment of APL came in 1983 and 1984 when two case reports described the successful single agent use of 13-cis-retinoic acid, an ATRA prodrug that is itself a less-potent RAR agonist [23,25,26]. In 1985, a subsequent case report was published describing the case of a five-year-old girl with APL at Shanghai Children’s Hospital successfully treated with ATRA. The patient went into complete remission within three weeks of administration, leading to further clinical drug development [26]. Later case studies demonstrated that ATRA was effective in salvaging patients previously treated with 13-cis-retinoin, leading to its preferential use in larger subsequent studies [27]. In 1988, a Shanghai group published a study in which single-agent ATRA induced CR in a remarkable 23 of 24 patients [28]. The efficacy was confirmed by another clinical study with a larger cohort, in which CR was achieved in 72% of patients [29].

Nevertheless, remissions with single-agent ATRA are typically transient [30]. To achieve more-durable remissions, subsequent trials combined ATRA and chemotherapy in the induction phase and followed with ATRA maintenance, resulting in an improved 5-year disease-free survival of 74% [31] (Table 1). More recently, dual-targeted therapy consisting of ATRA and ATO achieved superior event-free survival (EFS) and overall survival (OS) compared to ATRA plus chemotherapy for patients with low-to-intermediate risk of APL in the phase 3 trial (APL0406) [32,33] (Table 1). The updated analysis showed excellent results, with participants having an EFS at 72 months of 96.6% in the ATRA-ATO group compared to 77.4% in the ATRA-chemotherapy group [34]. However, the optimal regimen for patients with high-risk cases remains a debated issue. Idarubicin and gemtuzumab ozogamicin are most commonly used with ATRA-ATO [35,36,37], whereas the APL15 trial showed that ATRA-ATO with hydroxyurea used to control leukocytosis has the same efficacy as ATRA-ATO plus chemotherapy, even in high-risk patients [38]. An advantage of this chemotherapy-free regimen is that it is relatively well-tolerated by patients unfit for chemotherapy due to cardiac dysfunction or severe comorbidities. Oral formulations of ATO have also been developed and appear to have similar tolerability and efficacy, promising a more accessible and convenient therapeutic regimen in the future [39]. Hence, the remarkable evolution of APL therapy over the decades has drastically changed APL from one of the most fatal to one of the most curable subtypes of AML.

## 6. Disruption of Retinoid Signaling in APL

The success of ATRA in the treatment of APL may be due in part to the singular role of dysregulated retinoic acid signaling in its pathogenesis. An epidemiological study implying a single rate-limiting event in APL based on the constant incidence over life [49] and leukemia development in PML-RARa transgenic mice [50,51] suggests that the PML-RARa translocation is necessary and sufficient for triggering leukemogenesis. APL cells have remarkably stable genomes with co-occurring mutations in only a handful of genes involved in cell signaling and proliferation, including FLT3, WT1, NRAS, and KRAS, and noncoding regions within the intron of WT1 [52,53,54].

A large number of reviews have described the molecular pathogenesis of APL. In brief, the PML-RARa oncoprotein forms homodimers via the PML coiled-coil domain, interacts with RXR [55], and competes with RARα for binding to RARE within the target genes. Unlike wild-type RAR/RXR, the PML-RARa fusion receptor has a much higher affinity for corepressors and a lower sensitivity to physiological concentrations of ATRA, resulting in the constitutional repression of target genes. Consequently, PML-RARa/RXR acts in a dominant negative fashion, resulting in maturation arrest and the proliferation of myeloid progenitors and promyelocytes. PML-RARa also recruits the DNA-methylating enzymes DNMT1 and DNMT3a, leading to transcriptional repression through the hypermethylation of the promoter regions of target genes [56]. Furthermore, a genome-wide analysis of the PML-RARa/RXR binding site using Chip-seq in an APL cell line (NB4) and primary APL cells revealed that the PML-RARa /RXR dimer interacts with and represses a broader range of promoter regions than its RARa /RXR counterpart [57], likely contributing to the oncogenic action of the fusion protein [55]. The same group and others have also recently demonstrated that PML/RARa exerts transactivating functions through directly binding to target genes such as growth factor independent 1 transcriptional repressor (GFI-1), which is mediated by the recruitment of P300 and HDAC1 and the formation of super-enhancers [58,59]. Additionally, PML-RARa interferes with the formation of PML nuclear bodies, which blunts p53 signaling and leads to the enhanced self-renewal of leukemic cells [60]. The complex transcriptional dysregulation of extended target genes and disorganized PML nuclear bodies contributes to the differentiation block, aberrant self-renewal, and impaired apoptosis in APL cells.

## 7. Mechanisms of Action of ATRA

Mechanistically, ATRA has dual effects on the PML-RARa fusion protein: first, ATRA induces the transactivation of target genes. PML-RARa is not responsive to the physiological level (10^−10^–10^−9^ M) of ATRA, but pharmacological doses (10^−6^–10^−7^ M) of ATRA induce a conformational change in PML-RARa (followed by dissociation with corepressors including HDACs [61] and DNMTs [62]), and the recruitment of co-activators such as HAT, which allows for the reactivation of silenced target genes, thereby resuming differentiation. Transcriptomic analyses have revealed that ATRA treatment rapidly up-regulates many transcription factors related to hematopoiesis, including the C/EBP family in NB4 cells [63]. In addition, genome-wide analyses of epigenetic change in NB4 treated with ATRA also clarified the increase in H3 acetylation at the PML-RARα target genes [55]. These reports support the fact that that ATRA converts PML-RARa from a constitutive repressor into a transcriptional activator and reactivates the target genes. The other mechanism is the ATRA-induced degradation of PML-RARa through the ubiquitin/proteasome system [64]. The ATRA-induced degradation of its receptor is considered a general feature of nuclear receptor signaling, which provides a negative feedback loop of RA on its receptors [65]. Mutations in the ligand-binding domain of RARa are known to confer resistance to ATRA by reducing the binding affinity [66,67,68]. These mutations are found in a third of patients who relapsed after ATRA treatment and are associated with a reduced response to post-relapse salvage therapy [69]. Furthermore, the presence of an internal tandem duplication (ITD) in the FLT3 gene, an adverse risk factor for worse outcomes in patients treated without arsenic trioxide (ATO), blunts ATRA-induced PML-RARa degradation [70]. Although the two modes of action are generally coupled, some experimental data show that the degradation of PML-RARa (rather than the differentiation of leukemic cells) is primarily responsible for clinical response to ATRA treatment. In a PML-RARa transgenic mice model, low-dose retinoic acid triggers differentiation but does not fully degrade PML-RARa, and thus fails to eradicate leukemia-initiating cells [71]. Similarly, the rare promyelocytic leukemia zinc finger (PLZF)-RARa-driven APL cells terminally differentiate but are never cleared on RA therapy [71]. Interestingly, etretinate, a synthetic retinoid, triggers target gene activation and efficient APL cell differentiation at levels comparable to ATRA but does not efficiently degrade PML-RARa. Etretinate fails to abolish the capacity of PML-RARa-transformed cells to initiate leukemia, indicating that sustained PML-RARa protein is necessary for the self-renewal of leukemic cells [72]. These results suggest the induction of differentiation is insufficient to eradicate APL cells and that the degradation of PML-RARa is also necessary. ATO also triggers PML-RARa degradation [73], providing a rationale for the dramatic synergistic effect observed with the combination of ATRA and ATO [74].

## 8. Exploring the Activity of Retinoids in Non-APL AML

The remarkable success of ATRA therapy in APL, together with the preclinical data showing that ATRA induces transcriptional activation in most AML cell lines in tissue culture, encouraged the undertaking of numerous clinical trials investigating the benefit of adding ATRA to standard chemotherapy in non-APL AML. Contrary to expectations, the results from those trials were disparate from one another, and in sum, did not demonstrate clear benefit. In an initial phase 3 trial that included 242 elderly patients with AML, adding ATRA to induction and consolidation chemotherapy was associated with a statistically significant improvement in remission rate and OS [75]. However, a sub-analysis for predictive biomarkers revealed that the benefit of ATRA was restricted to the cases with an NPM1 mutation without FLT-ITD [76]. In contrast, the MRC AML 12 trial randomized 1075 younger patients with non-APL AML and high-risk MDS to induction therapy with or without ATRA and found that the addition of ATRA did not affect remission rate or OS. An absence of benefit was observed for the entire cohort and specific subgroups, including AML with mutated NPM1, FLT3-ITD, or CEBPα [2,3] (Table 1).

## 9. Retinoids and Rexinoids for the Treatment of Non-Leukemic Malignancies

The use of retinoids as anti-cancer therapy was also explored in other, non-leukemic malignancies. Neuroblastoma, a solid tumor of the peripheral nervous system, is the most common cancer diagnosed in infancy. High-risk neuroblastoma is a particularly aggressive form of the disease and is associated with poor prognosis. In vitro, ATRA induces differentiation in neuroblastoma cells, leading to the development of retinoids as a targeted, differentiating agent in the treatment of neuroblastoma [77]. Moreover, 13-cis-retinoic acid (isotretinoin) is an endogenous retinoic acid isoform that is less abundant and less potent than ATRA, though it is converted to ATRA in vivo. Nonetheless, 13-cis-retinoic acid has a favorable pharmacologic safety profile that led to its development as a targeted maintenance therapy in children with neuroblastoma [78]. Clinical studies of retinoids in neuroblastoma culminated in the pivotal Children’s Cancer Group study (CCG-3891), which found that isotretinoin significantly improved the event-free survival rate of children with high-risk neuroblastoma [42] (Table 1). Isotretinoin is now part of the standard-of-care maintenance therapy for high-risk neuroblastoma. There remains interest in building upon this success with alternative retinoids or combining them with other differentiating agents such as herbimycin A [77,79].

Bexarotene is a synthetic rexinoid that is approved by the Food and Drug Administration (FDA) for the treatment of cutaneous T-cell lymphoma, a subtype of peripheral T-cell lymphoma, in the relapsed/refractory setting [41] (Table 1). In the context of CTCL, bexarotene inhibits proliferation, induces apoptosis, and may even trigger the differentiation of malignant T cells [80], though its mechanism is less clearly defined than that of other retinoid-responsive malignancies.

## 10. Retinoids for the Prevention of Non-Hematologic Cancer

Several epidemiologic studies have suggested an inverse association between dietary vitamin A and cancer incidence. A study in 2000 found that a high nutritional intake of vitamin A and carotenoids was associated with a reduced risk of lung cancer in non-smoking women [81]. Another study published in 1999 using data from the Health Study found that women with a higher intake of vitamin A and several other vitamins had a lower incidence of post-menopausal breast cancer [82]. These observational studies inspired prospective, interventional trials that aimed to use retinoids as chemoprevention to reduce the incidence of a variety of cancers. Topical retinoids have been used with some success to prevent actinic keratosis—a precursor to non-melanoma skin cancer—but their systemic use as chemoprevention is not well studied. Studies of retinoids as a chemoprophylaxis against the development of head and neck cancer, breast cancer, and hepatocellular carcinoma have yielded mixed results [83]. The largest study, known as the CARET (Carotene and Retinol Efficacy Trial) study, found that a combination of retinyl palmitate (a retinoid) and beta-carotene actually increased the risk of lung cancer in smokers and asbestos-exposed workers [84]. Overall, these disappointing results have reduced enthusiasm for the use of retinoids as a chemoprophylaxis, though there are still some ongoing efforts to translate these theoretical benefits (NCT00201279).

## 11. ATRA Metabolism in Vivo

The reasons for retinoids’ failure to improve outcomes outside of APL and neuroblastoma are complex but are illuminated by a deepened understanding of retinoid metabolism and homeostasis. Continuous oral ATRA monotherapy induces only temporary remissions, with durations often being less than six months [30]. Researchers have speculated that these remissions are short-lived because of the rapid decline in steady-state ATRA concentrations that occur within days of the initiation of the drug [85,86]. A liposomal formulation of all-trans retinoic acid (L-ATRA) with better pharmacokinetic properties and higher maintained ATRA concentrations [87,88] led to sustained molecular complete remissions (CRs) with a median duration of 6.4 years in 10 out of 26 responders with low-risk APL [89]. Consistent with this result, experimental data demonstrated that only high concentrations of ATRA can fully degrade PML-RARa protein and eradicate leukemia-initiating cells in a PML-RARα transgenic mice model [71]. Collectively, this clinical and experimental evidence underscores that the levels of ATRA reaching leukemic cells are critical for complete eradication.

The balanced synthesis of ATRA by ALDH1A enzymes and metabolism by CYP26 enzymes, as well as other regulating enzymes, maintain ATRA homeostasis [90,91,92]. ATRA induces its catabolism by stimulating the up-regulation (up to a thousand-fold) of CYP26A1 mRNA expression in human hepatocytes, suggesting that its auto-induced catabolism is responsible for the accelerated clearance of ATRA and progressive decline of plasma ATRA concentrations in patients on continuous oral ATRA treatment [93,94]. Intermittent ATRA dosing schedules may be advantageous over continuous dosing schedules because they minimize the induction of auto catabolism [95]. Notably, systemic RA concentrations do not reflect RA homeostasis in specific tissues due to an intra-organ expression of CYP26 enzymes, which regulate RA levels locally [96]. The local production of the CYP26 enzyme within the bone marrow microenvironment may provide a protective niche for leukemic cells and contribute to leukemic cells acquiring resistance to ATRA.

Recent reports have implicated that the feedback mechanisms that control RA signaling are essential in regulating hematopoiesis within the bone marrow microenvironment. Although hematopoietic stem cells (HSC) rapidly differentiate in liquid culture, the co-culture system has shown that the human bone marrow-derived stromal cells are capable of metabolizing retinoids through CYP26A1 and CYP26B1 expression, thereby preserving the primitive phenotype and self-renewal capacity of HSCs through the maintenance of a low retinoid in an environment [97]. Similarly, pharmacological doses of ATRA exhibit activity against non-APL AML cells in liquid culture; however, stromal cells, which also up-regulate their CYP26 expression upon exposure to ATRA, blocked the pro-differentiation effect of ATRA on APL and AML cell lines as well as CD34^+^CD38^−^ALDH^int^ leukemic cells from primary samples, which were rescued by CYP26 inhibition [98]. These experimental data suggest that HSCs and leukemic cells are protected from RA signaling by CYP26 activity in the bone marrow niche, which provides a plausible explanation as to why clinical studies failed to demonstrate any benefit from the addition of ATRA to treatment regimens in non-APL AML [2,99]. Tamibarotene and IRX195183 are CYP26-resistant synthetic retinoids that primarily function as RARα agonists. Unlike ATRA, these synthetic retinoids maintain their pro-differentiating activity on APL cells in co-culture with bone marrow stromal cells [6]. Thus, CYP26-resistant synthetic retinoids have the potential to bypass the stromal-mediated protection and fully exert an anti-leukemic effect via the RARα-mediated differentiation of leukemic cells.

## 12. Synthetic Retinoids

**Figure 1:**
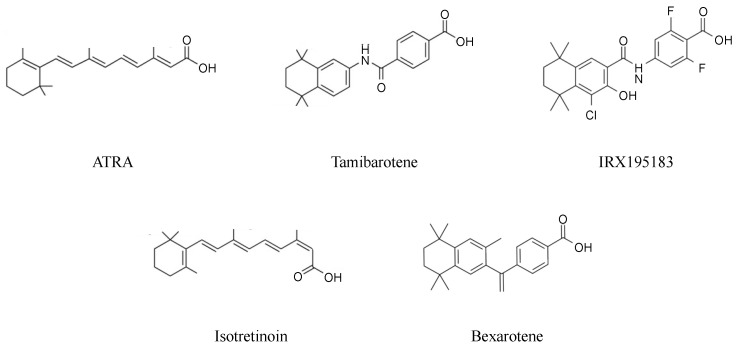
Molecular structures of ATRA and synthetic retinoids.

Building on the success of ATRA, many synthetic retinoids have been developed and evaluated for enhanced anti-tumor therapeutic potential [100,101] (Figure 1). Synthesized at the University of Tokyo in 1984, tamibarotene (formerly known as Am80) is a selective retinoid with a high affinity for RARa, resulting in greater potency as a differentiating agent [100] but a minimal affinity for RARγ [4]. It bears two methyl groups at C-4 that render it resistant to CYP26-mediated catabolism [6,102]. Thus, tamibarotene has a favorable pharmacokinetic profile with more sustained plasma drug levels than ATRA [46,103]. Furthermore, this agent is associated with a lower incidence and severity of cutaneous reactions, presumably related to its lower affinity for RARγ, mainly expressed in the dermal epithelium [104]. In Japan, tamibarotene has been studied in several clinical studies as a potentially superior alternative to ATRA. A prospective multicenter phase 2 study evaluated tamibarotene as a single agent in 24 patients who had relapsed after ATRA-containing treatment. Although those patients generally have difficulty in achieving a second CR with a second course of ATRA alone [105], tamibarotene achieved CR in 14 patients (68%) in this study, and four of them, after subsequent consolidation chemotherapy, have remained in CR for more than four years without stem cell transplantation [46,106] (Table 1). These results imply that tamibarotene overcomes the mechanisms that confer resistance to ATRA in vivo, including CYP26-mediated protection in bone marrow niche. Based on these results, tamibarotene was approved in Japan for the treatment of relapsed APL. A subsequent phase 3 study compared tamibarotene with ATRA as a maintenance therapy for newly diagnosed APL patients [47] (Table 1). An updated long-term analysis showed a statistically significant difference in relapse-free survival (RFS) of seven years between the ATRA group (84%) and the tamibarotene group (93%). When the analysis was restricted to high-risk patients, RFS at seven years was 62% in the ATRA group and 89% in the tamibarotene group, suggesting the potential role of tamibarotene as a maintenance therapy, especially in high-risk cases. Although the utility of tamibarotene is uncertain in the ATO era, the optimal management of patients relapsing after ATRA and ATO has been an unresolved issue. In this context, a multicenter phase 2 clinical study was conducted to evaluate the efficacy of tamibarotene in the relapsed/refractory setting. Tamibarotene as a single agent achieved CR/CRi in 64% of the cases, including molecular CR in 21%, although subsequent relapse occurred within six months in most cases. This result suggests that tamibarotene has significant activity in substantial patients with relapsed APL after ATRA and ATO treatment [107], and a more significant role in the cases that acquire PML mutations associated with the resistance to ATO [108,109].

IRX195183 (formerly known as NRX195183) is another promising CYP26-resistant synthetic retinoid. This agent is an exclusive RARα agonist, whereas ATRA is a pan-RAR agonist with relatively similar binding affinities for all three RAR isotypes [6]. The interplay between RARs and their ligands in normal hematopoiesis is complex and depends upon the differential stage of hematopoietic cells and physiological levels of retinoids [19]. The RARγ pathway appears to promote self-renewal of hematopoietic stem cells, while the RARα pathway promotes the myeloid differentiation of committed myeloid progenitors [110]. Consistent with this, a study using selective RAR agonists revealed the differentiation of AML cells appears to be mediated by mainly RARα signaling and only minimally by RARβ and RARγ [6]. Intriguingly, a study revealed that IRX195183 efficiently induced myeloid differentiation and the loss of the self-renewal of AML1/ETO-transformed progenitors, whereas ATRA potentiated the clonogenicity of them, probably through RARγ activation [5]. Transactivation studies revealed that a 0.24 nM level of ATRA was needed to transactivate RARγ, whereas 19.3 nM was needed for RARα, indicating that a substantially lower level of ATRA is sufficient for the activation of RARγ [111]. As described above, exogenous ATRA up-regulates CYP26 activity in bone marrow stromal cells, rendering the bone marrow niche a retinoid-deficient environment [6]. As a result, ATRA levels might fall below the threshold necessary for the transactivation of RARα but sufficient for RARγ transactivation, leading to the enhanced self-renewal of leukemic cells [5]. In this context, RARa-selective and CYP26-resistant retinoids may be preferable differentiation agents for AML cells. A phase 1 study of IRX195183 in patients with MDS and AML was recently conducted and showed that IRX195183 is safe and potentially effective in a subset of cases [112]. The clinical benefit of IRX195183 as a single agent or in combination with other drugs needs to be investigated in further studies. These synthetic retinoids could expand the application of retinoid-based therapy in both APL and non-APL AML.

## 13. Renewed Promise for Retinoids in Non-APL AML

More recent studies have tried to identify specific genetic subgroups, which may have greater retinoid sensitivity through functional RAR signaling [110]. AML with an overexpression of ecotropic viral integration site 1 (EVI-1) occurs in approximately 10% of patients with AML and is associated with poor prognosis. A study using AML cell lines showed that EVI-1 enhanced the transcriptional response to ATRA [113], and another study using primary samples showed that ATRA induces differentiation and reduces the clonogenic capacity for most of primary EVI-1-positive AML cells in vitro and in vivo, whereas other subtype AML cells lack such a response [114]. IDH mutations are discovered in approximately 15% of AML cases [115] and result in the production of the oncometabolite (R)-2-hydroxyglutarate (2-HG). Moreover, 2-HG is a competitive inhibitor of multiple α-ketoglutarate-dependent dioxygenases such as the TET family of DNA demethylases and the Jumonji family of histone demethylases, including lysine-specific demethylase 1 (LSD1) [116]. Furthermore, 2-HG dysregulates differentiation programs in leukemic cells through DNA and histone hypermethylation for several genes involved in RA signaling pathways [117,118]. A laboratory experiment revealed that IDH1-R132H mutation leads to the specific gene signatures enriched in myeloid differentiation and retinoid responsiveness. IDH1-mutated cell lines and IDH1-mutated primary cells are more sensitive to ATRA-induced differentiation and apoptosis than IDH1 wild-type cells in vitro and in vivo [119]. These experimental data suggest that EVI-1 overexpression and IDH1 mutation are possible biomarkers for ATRA sensitivity, although this assumption has not been tested in clinical trials.

The crosstalk between LSD1 and the ATRA-driven differentiation pathway has been well investigated. LSD1 is a mono- and di-methyl lysine demethylase targeting lysine 4 of histone H3 [120] and regulates hematopoietic myeloid differentiation as a transcriptional corepressor, partly through the interaction with GFI-1, which is a pro-differentiation transcription factor [121]. LSD1 is commonly overexpressed in AML [122] and is required to maintain AML cells [123]. Intriguingly, the inhibition of LSD1 potentiated an ATRA-driven differentiation of AML cell lines and primary cells together with the transcriptional activation of RAR target genes and also diminished the engraftment of primary AML samples in xenotransplantation models [7,124], suggesting that LSD1 is a promising target in combination with ATRA. Initially, the synergistic activity of this combination was thought to depend on the inhibition of LSD1 enzymatic activity; however, recent mechanistic studies have demonstrated that dissociation with LSD1 and GFI-1 is rather critical to sensitize AML cells to ATRA-driven differentiation [125]. Based on these laboratory results, phase 1 clinical trials of the LSD1 inhibitor tranylcypromine combined with ATRA have been conducted for patients with refractory/relapsed AML and MDS [43,44] (Table 1). The results showed an overall response rate of approximately 20% and a clinical benefit rate of roughly 40%. In one trial, gene signatures enriched in responders suggested more quiescent and less proliferative leukemic cells. In contrast, those increased in non-responders suggested a more proliferative phenotype characterized by mTOR signaling and MYC-regulated genes [43]. This evidence supports the further investigation of this combination strategy with more suitable patient selection and/or novel LSD1 inhibitors.

Super-enhancer (SE) is a large and highly active enhancer region that regulates essential genes to define cell identity and cell state [126,127]. More recently, SE analysis of AML patients has identified a novel subgroup with a strong SE in the RARα locus, resulting in a high level of RARα mRNA in leukemic cells, which accounted for approximately 25% of the samples analyzed and was not found in normal CD34-positive cells [8]. Intriguingly, this feature predisposes leukemic cells to an extremely high sensitivity to tamibarotene. Preclinical data showed that tamibarotene selectively induced RARα target genes such as ITGAX, ITGAM, and CD38, and inhibits the proliferation in in vitro and patient-derived xenograft (PDX) models of leukemic cells with high RARα, but not in RARα-low AML cells. Additionally, gene expression and enhancer responses to tamibarotene in RARα-high AML cells are like those of APL cells to retinoids. These results suggest a model where SE-induced over-expression of RARα results in an unbalanced excess of unliganded RARα, favoring the more repressive control of the myeloid differentiation program, as the PML-RARA fusion protein does in APL. Tamibarotene efficiently converts RARα from a repressive form to a transcriptional activator and reactivates differentiation pathways in RARα-high AML cells. Thus, a SE at the RARα locus could be a promising biomarker to select patients for tamibarotene treatment. In this study, the RARα-high PDX model was treated with tamibarotene or ATRA with doses corresponding to human exposure observed in the clinic. Intriguingly, tamibarotene showed better tumor reduction and more prolonged survival than ATRA, indicating that tamibarotene has higher potency against RARα-high AML cells, consistent with previous clinical studies showing the superiority of tamibarotene over ATRA in APL [47]. Based on these preclinical results, a biomarker-directed phase 2 trial of tamibarotene was conducted in AML and MDS patients with high RARα expression [128]. High mRNA expression was identified in approximately 30% of screened patients, and tamibarotene as a single agent achieved biological and clinical activity in 43% of the enrolled patients. Furthermore, synergistic activity with tamibarotene and hypomethylating agents (HMAs) such as azacitidine and decitabine was evaluated in RARα-high AML in a preclinical model [129]. This combination showed synergistic anti-proliferative effects in vitro and more profound and durable responses in PDX models than either agent alone, providing a rationale for designing a clinical trial of this combination strategy. In this context, a phase 2 study of tamibarotene and azacitidine combination was conducted in 51 newly diagnosed unfit AMLs including both RARα-high (N = 22) and RARα-low patients (N = 29), which were prospectively identified through real-time quantitative PCR assay (Table 1) [48]. Consistent with previous preclinical results, complete remission was much higher in RARα-high cases than in low cases (61% vs. 32%). The median time to initial complete response was rapid, at 1.2 months, and the median duration of composite complete remission was 10.8 months in RARα-high cases. Furthermore, exploratory RNA-seq analysis revealed that RARα-high patients show higher monocytic gene expression signatures than RARα-low patients, characterized by higher MCL-1 and lower BCL-2 expression. This finding is supported by TCGA non-APL AML datasets, demonstrating that approximately 70% of RARα-high patients show high monocytic gene expression signatures [48]. Notably, the BEAT AML dataset revealed that RARα-high samples show much lower ex vivo sensitivity to venetoclax than RARα-low samples [130], which is consistent with the accumulating reports of the association of monocytic AML with resistance to venetoclax [131,132]. Collectively, these results indicate that RARα-high AML cases are enriched for monocytic features associated with resistance to venetoclax-based therapies, and instead they are highly sensitive to tamibarotene and azacitidine combination. Currently, the phase 3 study of tamibarotene and azacitidine combination therapy for RARα-high high-risk MDS patients (NCT04797780), and a phase 2 study of tamibarotene plus venetoclax/azacitidine for newly diagnosed RARα-high AML patients (NCT04905407), are ongoing (Table 1).

## 14. Conclusions and Future Perspectives

ATRA was the first successful targeted cancer therapy, and it established a new paradigm for cancer treatment. Key to its success was the fact that it was first deployed in the treatment of a narrowly defined genetic subtype of AML. It is certainly serendipitous that the only genetically defined subtype of AML in the 1980s happened to be exquisitely responsive to ATRA, a retinoid that had been discovered 30 years earlier. Subsequent efforts to apply retinoids more broadly in the treatment and prevention of other cancers were disappointing, which is perhaps unsurprising in retrospect. Continued research has illuminated the reasons for these failures, including the body’s tight control of retinoid homeostasis and the heterogeneity of cancers, only some of which are genetically predisposed to being susceptible to retinoids. Breakthroughs in the understanding of retinoid metabolism as well as the revolution in cancer genetics are now leading to more thoughtful applications of synthetic retinoids with superior pharmacokinetics and to more precisely defined subsets of cancer, renewing hope that the full potential of retinoids and differentiating agents more broadly have not yet been realized.

## Figures and Tables

**Table 1:** Summary of clinical trials of retinoids in APL and other malignancies.

Studies of Retinoids Leading to Approved Use for the Study Population					
Patient Population	Study Design	Retinoid Regimen	Response Rates	Survival	Reference
Newly diagnosed APL	Randomized controlled trial	Chemotherapy vs. ATRA + chemotherapy	CR 81% vs. 91%, *p* = 0.25	1-year EFS 50% vs. 79% at 12 months, *p* = 0.001	[40]
Newly diagnosed APL	Randomized controlled trial	ATRA + chemotherapy vs. ATRA + arsenic trioxide	CR 95% vs. 100%, *p* = 0.12	2-year EFS 86% vs. 97%. *p* = 0.02 for superiority	[32]
Advanced stage (stage IIB-IVB) CTCL	Phase IIb/III clinical trial	Bexarotene	ORR 55%, CR 13%	Relapse rate after response 36%, Median duration of response 299 days	[41]
High Risk Neuroblastoma	Randomized controlled trial	Isotretinoin maintenance vs. placebo	NA	3-year EFS 46% vs. 29%, *p* = 0.027	[42]
**Studies of Retinoids in Diseases for Which They Are Not Specifically Approved**					
**Patient Population**	**Study Design**	**Retinoid Regimen**	**Response Rates**	**Survival**	**Reference**
AML (Non-APL)	Randomized controlled trial	ATRA + chemotherapy vs. chemotherapy alone	ORR 83% vs. 84% (*p* = NS)	8-year OS was 33% vs. 30%; HR, 0.98 [0.85–1.14])	[2]
Relapsed/Refractory AML and MDS	Phase I	ATRA + Tranylcypromine	ORR was 30.8% (95% CI = 10.4–61.1)	OS 5.0 months (95% CI = 3.2–21.6)	[43]
Relapsed/Refractory AML	Phase I/II	ATRA + Tranylcypromine	ORR 20%	one-year OS 22%	[44]
Unfit, Newly diagnosed AML (Non-APL)	Phase II Randomized controlled trial	ATRA + Decitabine vs. Decitabine alone	ORR 21.9% *vs* 13.5%; OR, 1.80; 95% CI, 0.86–3.79; one-sided *p* = 0.06	Median OS 8.2 months vs. 5.1 (HR, 0.65; 95% CI, 0.48–0.89; two-sided *p* = 0.006)	[45]
APL relapsed after ATRA	Phase II	Tamibarotene	58% CR	Not reported	[46]
Newly Diagnosed APL	Randomized controlled trial	Tamibarotene vs. ATRA Maintenance	NA	4-year RFS was 87% vs. 58% (HR, 0.26; 95% CI, 0.07–0.95)	[47]
Newly Diagnosed AML (RARA positive)	Phase II	Tamibarotene + Azacitidine	CR/CRi rate of 61%	median OS was 8.4 months (95% CI: 5.2, 15.6)	[48]
**Ongoing Clinical Trials Exploring New Applications for Retinoids**					
**Patient Population**	**Study Design**	**Retinoid Regimen**	**Response Rates**	**Survival**	**Reference**
Newly diagnosed or Relapsed AML (Non-APL)	Phase I	ATRA + ATO + Realgar-Indigo naturalis formula	NA	NA	NCT05297123
Relapsed or Refractory AML (Non-APL)	Phase I	TAS1440 (Oral LSD1 inhibitor) + ATRA	NA	NA	NCT04282668
Newly Diagnosed AML (Non-APL, RARA positive)	Phase II	Tamibarotene + Azacitidine/Venetoclax	NA	NA	NCT04905407
Newly diagnosed Multiple Myeloma	Phase II	Daratumumab, Pomalidomide, Dexamethasone, and ATRA	NA	NA	NCT04700176
Newly Diagnosed MDS (RARA positive)	Phase III Randomized Clinical Trial	Tamibarotene + Azacitidine	NA	NA	NCT04797780
